# Sulforaphane Inhibits Osteoclastogenesis via Suppression of the Autophagic Pathway

**DOI:** 10.3390/molecules26020347

**Published:** 2021-01-12

**Authors:** Tingting Luo, Xiazhou Fu, Yaoli Liu, Yaoting Ji, Zhengjun Shang

**Affiliations:** 1 The State Key Laboratory Breeding Base of Basic Science of Stomatology (Hubei-MOST) & Key Laboratory of Oral Biomedicine Ministry of Education, School & Hospital of Stomatology, Wuhan University, Wuhan 430000, China; luotingting@whu.edu.cn (T.L.); fuxiazhou@whu.edu.cn (X.F.); liuyaoli2017@whu.edu.cn (Y.L.); 2 Department of Oral and Maxillofacial-Head and Neck Oncology, School and Hospital of Stomatology, Wuhan University, Wuhan 430000, China

**Keywords:** sulforaphane, osteoclastogenesis, autophagy, JNK signaling pathway

## Abstract

Previous studies have demonstrated that sulforaphane (SFN) is a promising agent against osteoclastic bone destruction. However, the mechanism underlying its anti-osteoclastogenic activity is still unclear. Herein, for the first time, we explored the potential role of autophagy in SFN-mediated anti-osteoclastogenesis in vitro and in vivo. We established an osteoclastogenesis model using receptor activator of nuclear factor kappa-β ligand (RANKL)-induced RAW264.7 cells and bone marrow macrophages (BMMs). Tartrate-resistant acid phosphatase (TRAP) staining showed the formation of osteoclasts. We observed autophagosomes by transmission electron microscopy (TEM). In vitro, we found that SFN inhibited osteoclastogenesis (number of osteoclasts: 22.67 ± 0.88 in the SFN (0) group vs. 20.33 ± 1.45 in the SFN (1 μM) group vs. 13.00 ± 1.00 in the SFN (2.5 μM) group vs. 6.66 ± 1.20 in the SFN (2.5 μM) group), decreased the number of autophagosomes, and suppressed the accumulation of several autophagic proteins in osteoclast precursors. The activation of autophagy by rapamycin (RAP) almost reversed the SFN-elicited anti-osteoclastogenesis (number of osteoclasts: 22.67 ± 0.88 in the control group vs. 13.00 ± 1.00 in the SFN group vs. 17.33 ± 0.33 in the SFN+RAP group). Furthermore, Western blot (WB) analysis revealed that SFN inhibited the phosphorylation of c-Jun N-terminal kinase (JNK). The JNK activator anisomycin significantly promoted autophagy, whereas the inhibitor SP600125 markedly suppressed autophagic activation in pre-osteoclasts. Microcomputed tomography (CT), immunohistochemistry (IHC), and immunofluorescence (IF) were used to analyze the results in vivo. Consistent with the in vitro results, we found that the administration of SFN could decrease the number of osteoclasts and the expression of autophagic light chain 3 (LC3) and protect against lipopolysaccharide (LPS)-induced calvarial erosion. Our findings highlight autophagy as a crucial mechanism of SFN-mediated anti-osteoclastogenesis and show that the JNK signaling pathway participates in this process.

## 1. Introduction

Throughout life, bone remodeling is precisely regulated by the balance between osteoblastic bone formation and osteoclastic bone resorption. This balance is often disturbed by diseases associated with excessive bone resorption, such as osteoporosis, rheumatoid arthritis (RA), periodontitis, and bone metastasis [1,2,3]. Osteoclasts, multinucleated cells that derive from the monocyte–macrophage lineage of hematopoietic stem cells, are responsible for bone destruction in these diseases. Therefore, osteoclasts are crucial targets in the treatment of such diseases.

Sulforaphane (SFN), a sulfur-containing compound, is derived from cruciferous vegetables like broccoli sprouts [4]. The properties of SFN that have been mostly studied include anti-tumor, anti-inflammatory, and anti-oxidative activities [5]. Furthermore, SFN is reported to have potential beneficial effects in the treatment of diabetes, RA, and osteosarcoma, possibly by activating the antioxidant response and inhibiting inflammatory responses [6,7,8]. Recently, SFN was found to exhibit bone anabolic effects resulting from anti-osteoclastogenic and osteoblastic stimulatory effects [9]. Therefore, SFN shows promising efficacy as a potential therapy against excessive bone resorption-related pathologies. Takagi et al. suggested that SFN inhibits osteoclastic differentiation and cell fusion [10]. However, the underlying mechanisms are still unknown.

Autophagy, a conserved lysosome degradation pathway, plays an adaptive role in cellular survival, differentiation, and homeostasis [11]. Autophagy is associated with various diseases, including cancers and infection. Recent studies have implicated changes in autophagy in osteoclast-related diseases such as osteoporosis and RA [12,13]. Therefore, the pharmacological regulation of autophagy could be helpful in treating these diseases. For instance, rapamycin (RAP), a classical autophagy inducer, can efficiently decrease osteolysis associated with bone metastases in breast cancer [14]. Moreover, autophagy-related genes (Atgs) and Beclin1, the necessary molecules for autophagy, play pivotal roles in osteoclastogenesis. Carl et al. suggested that the Atg5–Atg12 complex is also crucial for osteoclastic lysosome localization and bone resorption and that its deficiency could impair the formation of osteoclasts’ ruffled border, resulting in osteopetrosis [15]. Beclin1 can be activated as early as 2 h after receptor activator of nuclear factor kappa-β ligand (RANKL) treatment, and bone marrow macrophages (BMMs) from Beclin1 conditional deletion mice exhibit suppression of osteoclastogenesis [16]. Therefore, autophagy is believed to be a critical participant in osteoclastogenesis. Notably, SFN exhibits a modulatory role of autophagy which has been implicated in neurodegenerative diseases, tumor therapy, and diabetic kidney disease. It was reported that SFN could induce autophagy in neuron cells and breast cancer cells, while it plays a negative role in renal tubule cells and esophageal cancer [17,18,19,20]. However, whether autophagy plays a significant role in anti-osteoclastogenesis mediated by SFN remains unclear.

Herein, the potential role of autophagy in SFN-mediated anti-osteoclastogenesis was explored for the first time. We demonstrate the effects of SFN on bone resorption in mice with lipopolysaccharide (LPS)-induced erosion of the calvarial bone and we investigated the alterations of autophagy in this process.

## 2. Results

### 2.1. SFN Inhibited Osteoclastogenesis in a Dose-Dependent Manner

**Figure 1:**
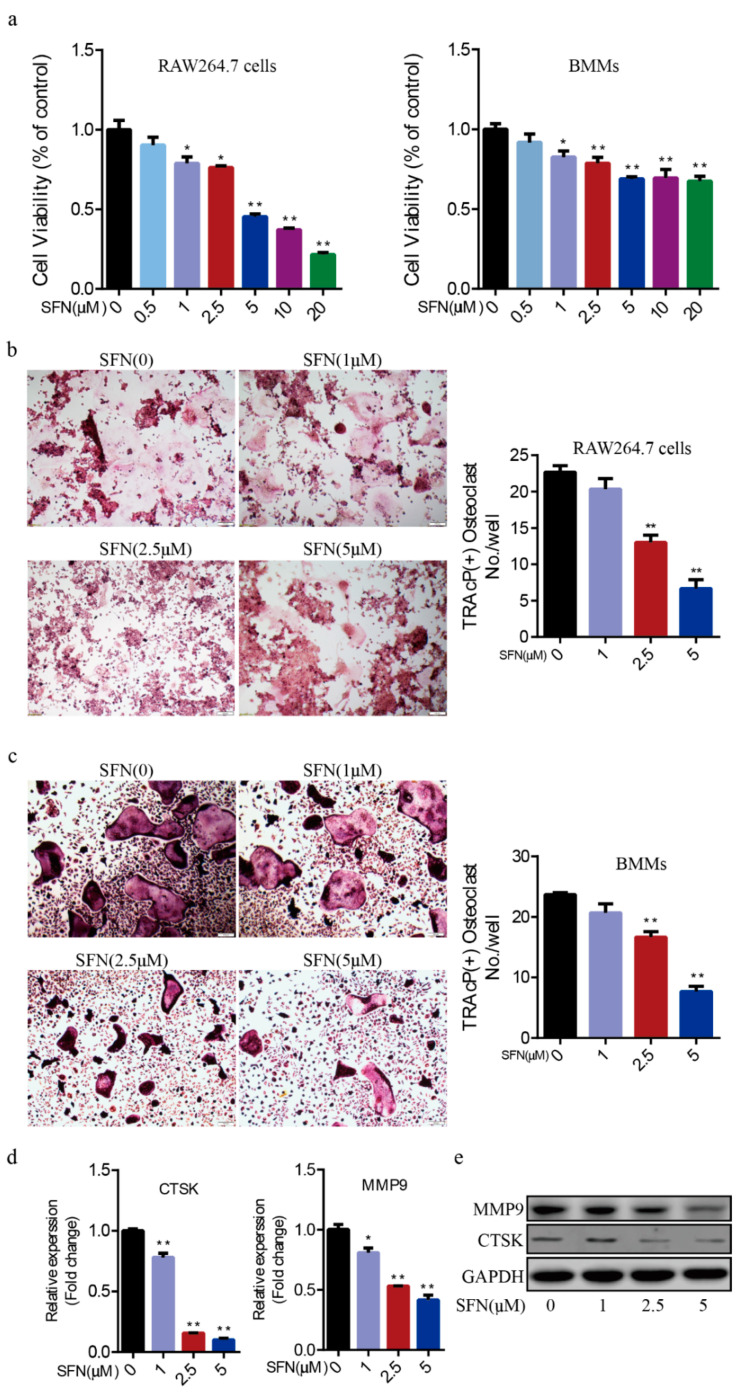
Sulforaphane (SFN) inhibited osteoclastogenesis in a dose-dependent manner. (**a**) The proliferative activity of RAW264.7 cells and bone marrow macrophages (BMMs) was detected by Cell Counting Kit-8 (CCK-8) assay after 24 h in the presence of the indicated concentration of SFN. For the osteoclastogenesis assay, RAW264.7 cells and BMMs were cultured with receptor activator of nuclear factor kappa-β ligand (RANKL, 50 ng/mL) and treated with varying concentrations of SFN (0, 1, 2.5, 5 μM) for 5 days. Representative images of osteoclasts differentiated from RAW264.7 cells (**b**) and BMMs (**c**) in each group and corresponding statistics. (**d**,**e**) Representative qRT-PCR and WB results showing the effects of SFN on osteoclastic matrix metalloproteinase 9 (MMP9) and cathepsin K (CTSK) expression. Values are mean ± SEM of three independent experiments; * *p* < 0.05, ** *p* < 0.01.

To explore the effect of SFN on the proliferative activity of pre-osteoclasts, we performed a Cell Counting Kit-8 (CCK-8) assay. We found that cell viability decreased along a gradient by treating RAW264.7 cells and BMMs with various concentration SFN (0, 0.5, 1, 2.5, 5, 10, 20 μM, Figure 1a). We cultured RAW264.7 cells and BMMs with RANKL (50 ng/mL) and treated them with various concentrations of SFN (0, 1, 2.5, 5 μM) for 5 days. Tartrate-resistant acid phosphatase (TRAP) staining showed that SFN decreased the number of osteoclasts in a dose-dependent manner (RAW264.7 cells: 22.67 ± 0.88 in the SFN (0) group vs. 20.33 ± 1.45 in the SFN (1 μM) group vs. 13.00 ± 1.00 in the SFN (2.5 μM) group vs. 6.66 ± 1.20 in the SFN (2.5 μM) group; BMMs: 23.67 ± 0.33 in the SFN (0) group vs. 20.67 ± 1.45 in the SFN (1 μM) group vs. 16.67 ± 0.88 in the SFN (2.5 μM) group vs. 7.67 ± 0.88 in the SFN (2.5 μM) group) (Figure 1b,c). Given the cytotoxicity of SFN, SFN at 2.5 μM, which has no obvious cytotoxicity, completely inhibited osteoclastogenesis. In addition, quantitative reverse-transcription polymerase chain reaction (qRT-PCR) and Western blot (WB) analyses showed that SFN suppressed the expression of both cathepsin K (CTSK) and matrix metalloproteinase 9 (MMP9) induced by RANKL (Figure 1d,e).

### 2.2. SFN Treatment Blocked the Autophagic Pathway in Pre-Osteoclasts

**Figure 2:**
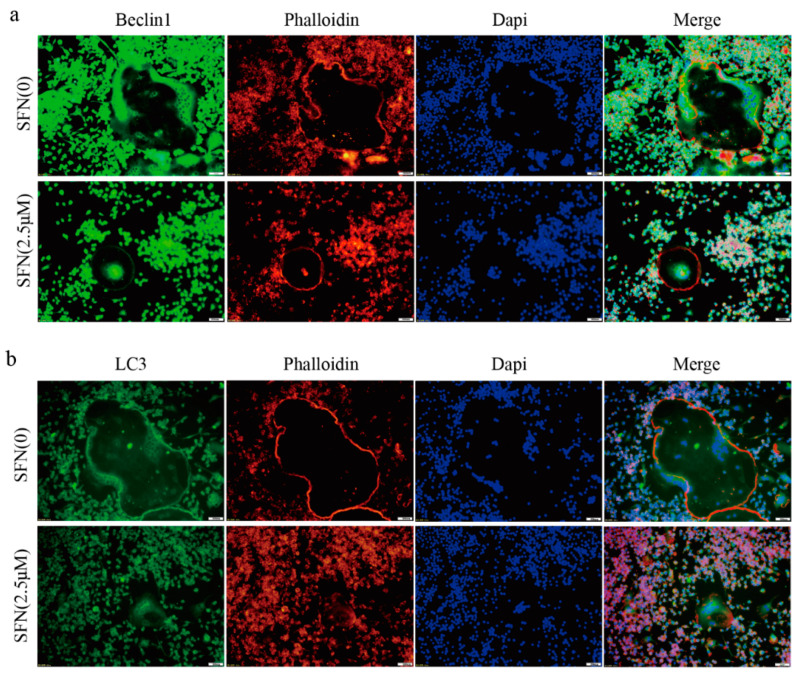
SFN inhibited the accumulation of autophagic light chain 3 (LC3) and Beclin1 within osteoclasts. RAW264.7 cells were induced by 50 ng/mL of RANKL for 5 days, and immunofluorescence (IF) analysis was performed. (**a**) Representative IF images locating LC3 (green) within the osteoclasts. (**b**) Representative IF images locating Beclin1 (green) within the osteoclasts. F-actin was stained with phalloidin (red). DAPI (blue) was used to visualize the nuclei.

**Figure 3:**
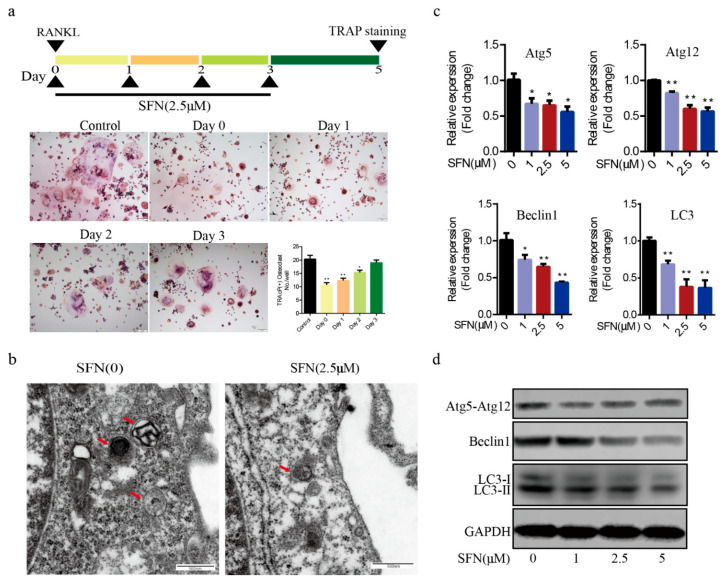
SFN treatment blocked the autophagic pathway in pre-osteoclasts. RAW264.7 cells were cultured with 50 ng/mL of RANKL for 5 days plus 2.5 μM SFN at the indicated time points. (**a**) Representative images showing osteoclastogenesis in each group and corresponding statistics. To explore the effect of SFN on autophagy in pre-osteoclasts, we stimulated RAW 264.7 cells with SFN for 2 h and then treated them with 50 ng/mL of RANKL for 24 h. (**b**) Representative images of autophagosomes (red arrow) as observed by TEM. (**c**,**d**) Representative qRT-PCR and WB results showing the effects of SFN on autophagic molecules (Atg5, Atg12, Beclin1, and LC3) expression. Values are mean ± SEM of three independent experiments; * *p* < 0.05, ** *p* < 0.01.

Subsequently, we investigated the precise mechanisms underlying SFN-mediated anti-osteoclastogenesis. Recent studies have shown that the activation of autophagy plays a vital role in osteoclast differentiation. Notably, obvious accumulation of light chain 3 (LC3)-Ⅱ and Beclin1 indicated by punctuated fluorescence within osteoclasts was suppressed by SFN treatment (Figure 2a,b). To explore the stage at which SFN mainly inhibited osteoclastogenesis, we exposed pre-osteoclasts to SFN at different time points (days 0, 1, 2 and 3). RAW264.7 cells were cultured with 50 ng/mL RANKL plus 2.5 μM SFN at the indicated time points. The results demonstrated that SFN treatment on day 0 efficiently inhibited osteoclastogenesis, whereas at later stages the inhibitory effect was limited (number of osteoclasts/well: 20.33 ± 1.45 in control group vs. 10.67 ± 0.88 on Day 0 group vs. 12.33 ± 0.88 in Day 1 group vs. 15.33 ± 0.88 on Day 2 group vs. 19.00 ± 1.00 on Day 3 group, Figure 3a). To explore the effect of SFN on autophagy in pre-osteoclasts, we stimulated RAW 264.7 cells with SFN for 2 h and then treated them with 50 ng/mL RANKL for 24 h. Compared with the control group, transmission electron microscopy (TEM) analysis showed a significant reduction in autophagosomes in the SFN (2.5 μM) group (Figure 3b). As shown in Figure 3c,d, SFN treatment significantly attenuated the transcriptional and translational levels of the autophagy markers LC3-II, Beclin1, and Atg5–Atg12.

### 2.3. Rapamycin Reversed SFN-Mediated Anti-Osteoclatogenic Effects

**Figure 4:**
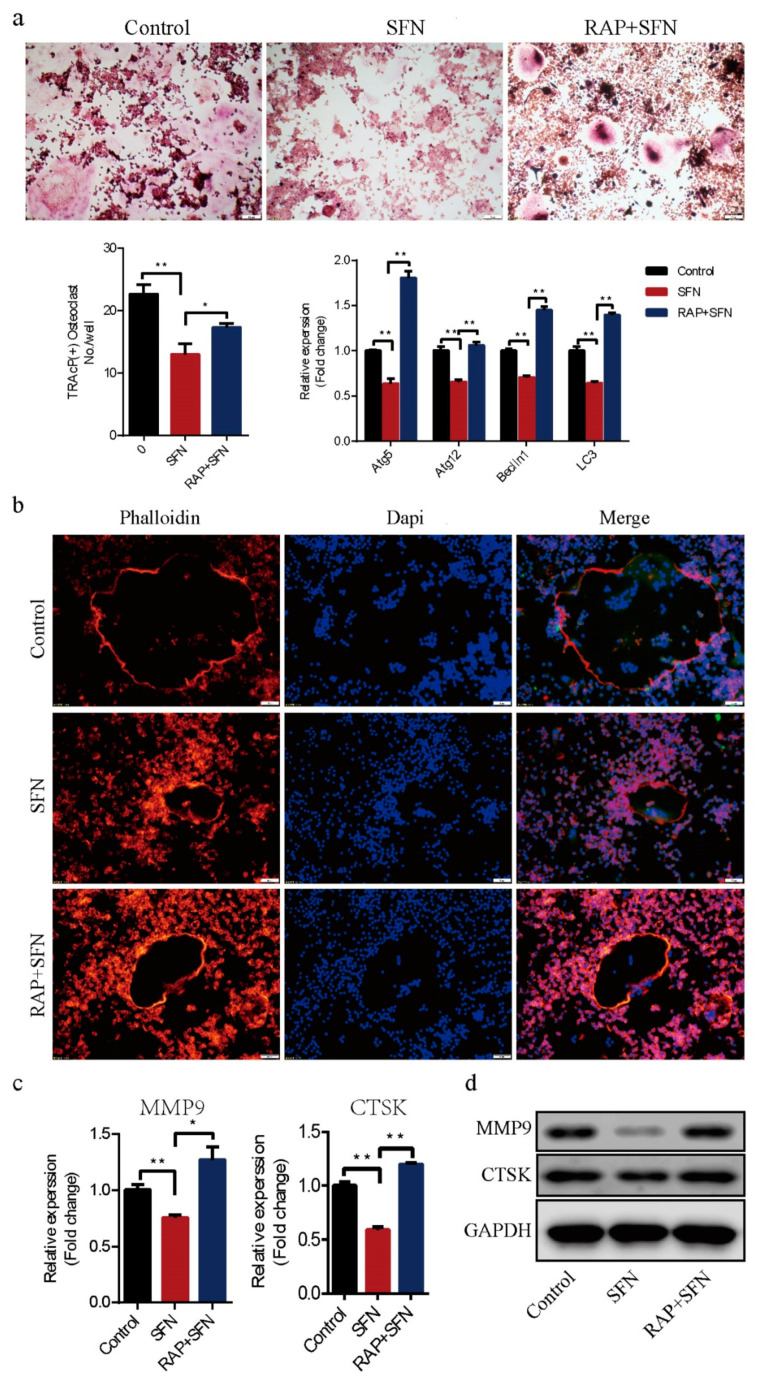

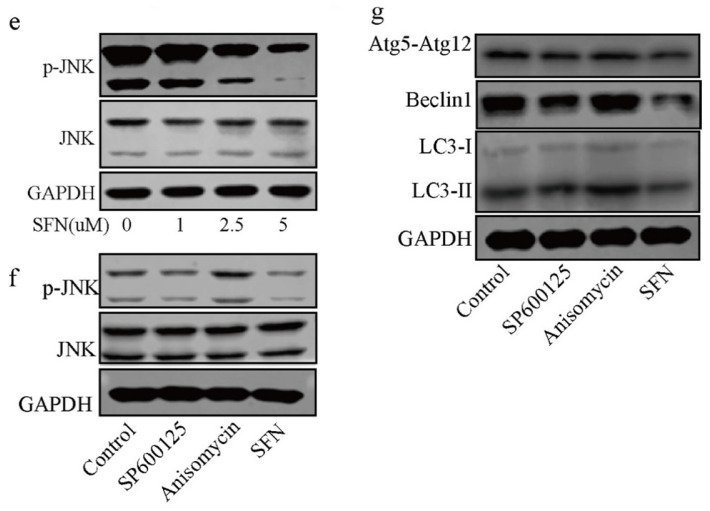
RAP reversed SFN-mediated anti-osteoclatogenic effects. Raw264.7 cells were cultured with 50 ng/mL of RANKL in the absence or presence of 2.5 μM SFN and 10 μM RAP for 5 days. (**a**) Representative images showing osteoclastogenesis in each group and corresponding statistics. (**b**) Representative images showing F-actin stained with phalloidin (red). DAPI (blue) was used to visualize the nuclei. (**c**,**d**) Representative qRT-PCR and WB results showing MMP9 and CTSK expression. To detect JNK signaling, RAW 264.7 cells were stimulated with SP600125. Anisomycin, or SFN for 2 h and then treated with 50 ng/mL of RANKL for 30 min. (**e**) WB results showing the activation level of JNK signaling after treatment with varying concentrations of SFN. (**f**) WB results showing the activation level of JNK signaling. To explore the role of JNK signaling in SFN-mediated inhibition of autophagy, we stimulated RAW 264.7 cells with SP600125, anisomycin, or SFN for 2 h and then treated them with 50 ng/mL of RANKL for 24 h. (**g**) WB results showing protein levels of autophagic molecules (Atg5, Atg12, Beclin1, and LC3). Values are mean ± SEM of three independent experiments; * *p* < 0.05, ** *p* < 0.01.

To investigate whether autophagy was involved in SFN-mediated anti-osteoclastogenesis, we used RAP to active autophagy in RAW264.7 cells. The cells were cultured with 50 ng/mL of RANKL in the absence or presence of 2.5 μM SFN and 10 μM RAP for 5 days. We found that RAP rescued the transcription of Beclin1, Atg5, Atg12, and LC3, which was inhibited by SFN (Figure 4a). RAP stimulation reversed SFN inhibition of osteoclastogenesis (number of osteoclasts/well: 22.67 ± 0.88 in the control group vs. 13.00 ± 1.00 in the SFN group vs. 17.33 ± 0.33 in the SFN+RAP group, Figure 4a). Similarly, SFN reduced the size of F-actin rings, whereas RAP reversed this reduction (Figure 4b). As expected, the expression of MMP9 and CTSK at the transcriptional and translational levels exhibited similar changes (Figure 4c,d). These results indicated that autophagy could be involved in SFN-mediated anti-osteoclastogenesis in vitro.

### 2.4. c-Jun N-Terminal Kinase (JNK) Signaling Pathway Appeared to Participate in SFN-Mediated Autophagy Inhibition in the Early Period of Osteoclastogenesis

To explore the role of JNK signaling in SFN-mediated autophagy inhibition in the early period of osteoclastogenesis, we stimulated RAW 264.7 cells with SP600125, anisomycin, or SFN for 2 h and then treated them with 50 ng/mL of RANKL for 30 min. As shown in Figure 4e, SFN suppressed the phosphorylation of JNK in a dose-dependent manner. To demonstrate the role of JNK signaling in autophagy during osteoclasogenesis, we treated RAW 264.7 cells with anisomycin, a JNK signaling activator, and SP600125, a JNK signaling inhibitor. WB results showed that anisomycin increased JNK phosphorylation, and SP600125 exerted a negative effect (Figure 4f). After stimulation with SFN for 2 h, RAW 264.7 cells were treated with 50 ng/mL of RANKL for 24 h. We found that the protein levels of LC3-II and Beclin1 were augmented after treatment with anisomycin, whereas SP600125 markedly decreased the expression of these proteins at the translational level (Figure 4g). Considering the suppression of autophagy in SFN-mediated anti-osteoclastogenesis, we suggest that SFN could inhibit autophagy via the suppression of JNK phosphorylation in the early period of osteoclastogenesis.

### 2.5. SFN Protected Against LPS-Induced Calvarial Bone Destruction in Mice

**Figure 5:**
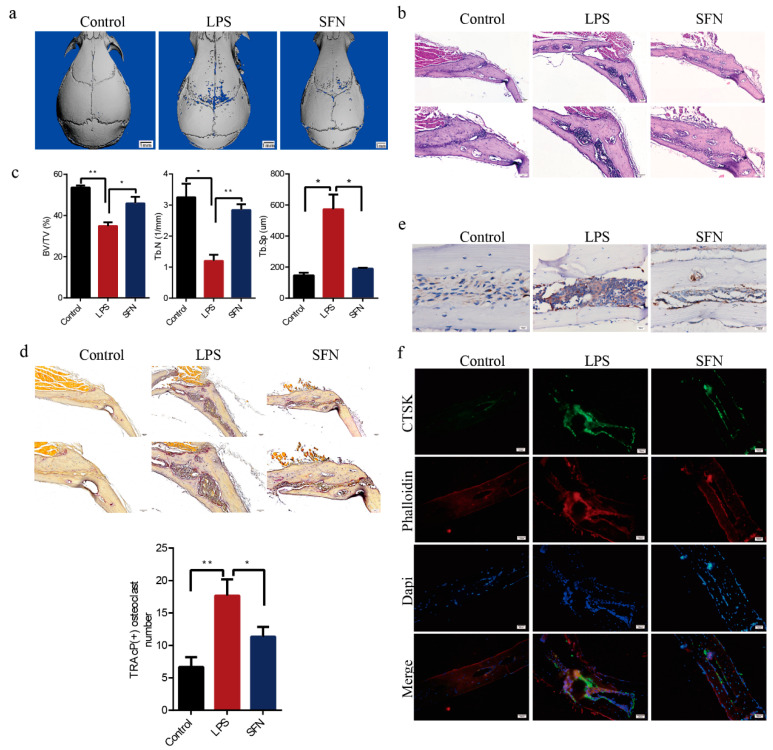
SFN protected against lipopolysaccharide (LPS)-induced calvarial bone destruction in mice. (**a**) Micro computed-tomography (CT) 3D reconstructed images. Calvarias were fixed, decalcified, dehydrated, embedded, and sectioned. (**b**) Representative hematoxylin-and-eosin (HE) staining images. (**c**) Microarchitectural parameter analysis: trabecular bone volume/total volume (BV/TV), trabecular number (Tb.N), and trabecular separation (Tb.Sp). (**d**) Representative tartrate-resistant acid phosphatase (TRAP) staining images showing osteoclastogenesis in each sample and corresponding statistics. (**e**) Representative IHC images locating CTSK. (**f**) Representative IF images of the distribution of CTSK (green). Phalloidin (red) indicates cytoskeleton and bone. DAPI (blue) was used to counterstain the nuclei. Values are mean ± SEM of three independent experiments; * *p* < 0.05, ** *p* < 0.01.

Mouse calvarial models were established to assess how well SFN could prevent pathological erosion. Microcomputed-tomography (CT) scanning and three-dimensional (3D) reconstruction revealed that LPS-injected calvarias had obvious surface erosion, while the simultaneous administration of SFN showed limited calvarial destruction (Figure 5a). Consistent with the microCT analysis, hematoxylin-and-eosin (HE) staining revealed the interruption of bone continuity in the LPS group, while the administration of SFN inhibited this effect. Therefore, we suggest that SFN could protect bone against destruction (Figure 5b). Trabecular bone volume (BV) /total volume (TV) and number (Tb.N) were higher in the SFN than in the LPS group, while trabecular separation (Tb.Sp) was clearly lower in the SFN group (Figure 5c). In addition, LPS-stimulated calvarias showed an increase in osteoclasts, while SFN significantly decreased the number of osteoclasts induced by LPS (Figure 5d). We also analyzed by immunohistochemistry (IHC) and immunofluorescence (IF) the expression of CTSK, an osteoclastic marker protein. CTSK staining was distributed at a higher intensity in the LPS group and was weakened in the SFN group. These results revealed that SFN could suppress osteoclastogenesis to prevent calvarial bone erosion.

**Figure 6:**
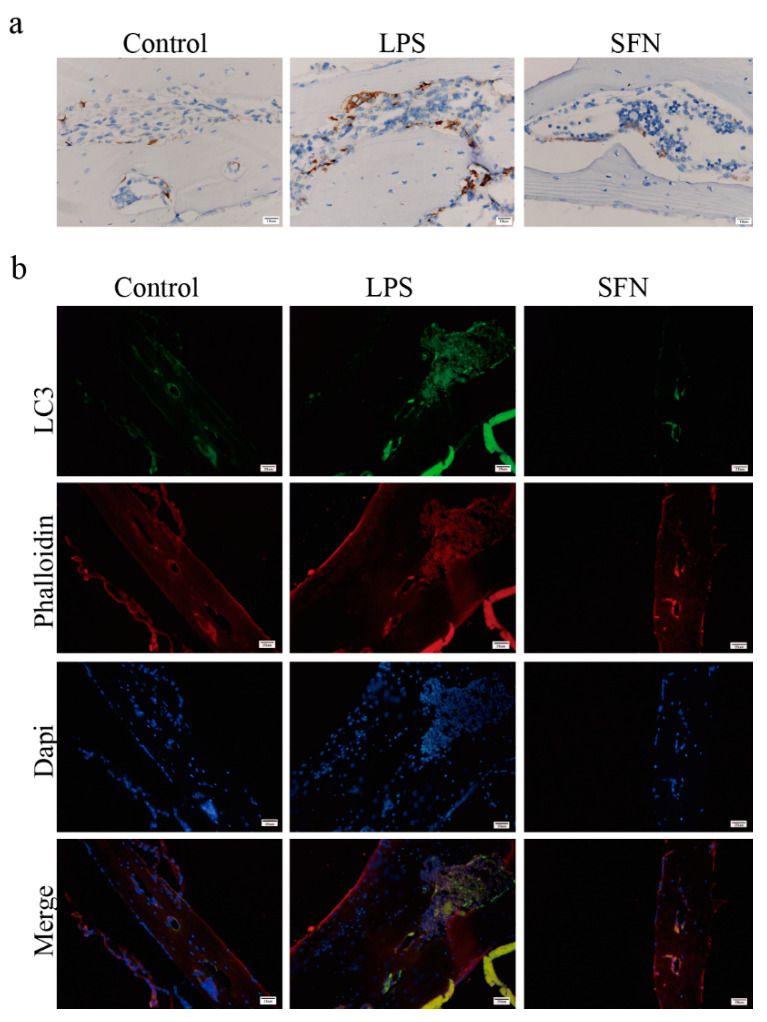
The administration of SFN attenuated autophagic LC3 expression in an LPS-induced mouse calvaria model. Calvarias were fixed, decalcified, dehydrated, embedded, and sectioned. (**a**) Representative IHC images locating LC3. (**b**) Representative IF images of the distribution of LC3 (green). Phalloidin (red) indicates cytoskeleton and bone. DAPI (blue) was used to counterstain the nuclei.

Subsequently, we examined the expression level of LC3 in vivo. As shown in Figure 6a, IHC analysis indicated that the expression of LC3 was induced in the LPS group compared with the control group in mice calvaria, while SFN suppressed this effect. IF analysis showed similar results (Figure 6b).

## 3. Discussion

SFN is an isothiocyanate derived from cruciferous vegetables. Over recent decades, extensive studies have focused on its antitumor activity at different tumor stages, from tumorigenesis to progression. As a pleiotropic compound, SFN is known to protect cells from deoxyribonucleic acid (DNA) damage and to modulate cell proliferation, apoptosis, angiogenesis, invasion, and metastasis in cancer [21,22,23,24]. Recently, it was reported that SFN could also be used to treat diseases associated with pathological bone resorption, such as bone metastasis in breast cancer, osteoporosis, and osteoarthritis [25,26,27]. In addition, Tomohiro Takagi et al. suggested that SFN plays a negative role in RANKL-induced osteoclastogenesis, but the underlying mechanisms need to be further investigated [10,28]. Autophagy is required during osteoclastogenesis and could be modulated by SFN. In this study, for the first time, we demonstrated that the suppression of the autophagy pathway is a significant mechanism by which SFN negatively regulated osteoclastogenesis.

SFN, a multipotent compound, has been reported to mediate autophagy in esophageal squamous cell carcinoma, pancreatic cancer, and neuronal cells [20,29,30]. SFN could inhibit autophagy in esophageal squamous cell carcinoma and renal tubule cells, while it promoted autophagy in neuronal cells [17,18,19,20]. As is well known, the level of autophagy varies in different cell types. We first investigated the effect of SFN on autophagy in pre-osteoclasts. Autophagy is characterized by three distinct stages: phagophore, autophagosome, and autolysosome. This process is regulated and executed by autophagy-related proteins such as the Atg system [31]. Beclin1 (Atg6) is responsible for forming an initial autophagosome complex; LC3 (Atg8), recruited by the Atg5–Atg12 complex, is essential for the formation and maturation of autophagosomes [32,33]. Herein, our data showed that SFN largely decreased the expression of these markers in RANKL-induced RAW264.7 cells, indicating that it interrupted autophagy. In addition, TEM observation showed that SFN also reduced the number of double-membraned autophagosomes. This confirmed that SFN could significantly inhibit autophagy in pre-osteoclasts.

Autophagy, which is involved in various biological cell processes such as proliferation, differentiation, and survival, principally plays an adaptive role in order to maintain organismal homeostasis [34,35,36]. Emerging evidence shows that autophagy might be a potential target for regulating osteoclastic differentiation. Particular conditions, such as starvation and high-glucose inflammation, could alter autophagy in pre-osteoclasts and influence osteoclastogenesis, leading to pathological changes [37,38,39]. Fortunately, autophagy can be pharmacologically regulated by certain agents. For instance, RAP, an inhibitor of mammalian target of rapamycin (mTOR) which negatively regulates autophagy, can induce autophagy [40]. Our data showed that SFN attenuated LC3-II accumulation in pre-osteoclasts, while RAP almost reversed this effect, thereby contributing to the recovery of osteoclastogenesis and the expression of osteoclast-related proteins (MMP9 and CTSK). Collectively, our data highlight the suppression of autophagy as a significant mechanism in SFN-mediated anti-osteoclastogenesis.

Mitogen-activated protein kinase (MAPK) signaling (involving JNK, p38, and extracellular signal-regulated kinase [ERK]) induced by RANKL is required for osteoclastic differentiation [41]. p38/ERK signaling is reported to play a dual role in the regulation of autophagy, acting as an activator or an inhibitor depending on the cellular context and particular conditions [42,43,44]. JNK signaling can induce autophagy and consequently contributes to cell survival, indicating a positive correlation between JNK signaling and autophagy [45]. Therefore, in this study, we focused on the role of the JNK signaling pathway in SFN-mediated anti-osteoclastogenesis. Our results showed that SFN treatment decreased JNK phosphorylation during osteoclastic differentiation. In addition, the activation of JNK signaling promoted the expression of autophagic proteins, while the inhibition of JNK phosphorylation suppressed such expression, which indicated that JNK signaling participated in the activation of autophagy during osteoclastogenesis. Considering the role of autophagy in SFN-mediated anti-osteoclastogenesis, we suggest that SFN could inhibit osteoclastogenesis by suppressing autophagy through the disruption of the JNK signaling pathway.

LPS can induce osteoclastic bone erosion by mediating the production of inflammatory factors such as interleukin-1 (IL-1) and tumor necrosis factor (TNF) [46,47]. In our study, we established a bone erosion model by subcutaneously injecting LPS into the center of mouse calvarias. Our results showed that SFN suppressed LPS-induced local calvarial erosion in vivo, supporting the possible application of SFN against diseases that feature inflammatory bone destruction. In addition, the number of osteoclasts was significantly reduced after treatment with SFN, indicating that osteoclasts were a target in SFN-mediated prevention of bone destruction. Furthermore, our data showed accumulation of LC3-II in LPS-stimulated calvarias.

Taken together, our results reveal for the first time that SFN inhibits RANKL-induced osteoclastogenesis in vitro and LPS-mediated bone erosion in vivo by suppressing autophagy. Furthermore, we demonstrated that the JNK signaling pathway participated in the activation of autophagy and SFN inhibited the phosphorylation of JNK in osteoclastogenesis. Taken together, we suggest that SFN could inhibit osteoclastogenesis by suppressing autophagy through the disruption of the JNK signaling pathway. Therefore, this study highlights SFN as a potent drug candidate for the prevention and treatment of bone loss-related diseases and shows that autophagy is a promising important mechanism of SFN-dependent anti-osteoclastogenic effect.

## 4. Materials and Methods

### 4.1. Reagents and Mice

RAW 264.7 cells were obtained from the China Center for Type Culture Collection (Shanghai, China). RANKL and M-CSF were purchased from R&D Systems (Minneapolis, MN, USA); α-modified Eagle’s medium (MEM) was from Hyclone (UT, USA). Fetal bovine serum (FBS) was obtained from Gibco (Gaithersburg, MD, USA). SFN (Cat. No. S4441) was purchased from Sigma-Aldrich (St. Louis, MO, USA). The primary antibodies to Atg12–Atg5 (Cat.No. 4180), Beclin-1 (Cat.No. 3495), LC3 (Cat.No. 3868), phospho-JNK (Cat.No. 9255), and JNK (Cat.No. 9252) were obtained from Cell Signaling Technology (MA, USA). The primary antibody for MMP9 (Cat.No. 10375-2-AP) was from Proteintech (Hubei, China). The primary antibody for CTSK (Cat.No. AP7381) was obtained from Abcepta (Jiangsu, China). GAPDH Mouse Monoclonal Antibody (2B8) HRP-conjugated (Cat.No. PMK053) and (FITC)-conjugated secondary antibodies (Cat.No. PMK-014-093) were obtained from Bioprimacy (Hubei, China). C57/BL6 mice (female; 8-week-old) were purchased from Beijing Vital River Laboratory Animal Technology (Beijing, China). TRAP staining kit, LPS, and rapamycin were purchased from Sigma-Aldrich (MO, USA). BCA protein assay kit was from Thermo Fisher Scientific (Rockford, IL, USA). PCR-related agents were from Takara (Tokyo, Japan).

### 4.2. Preparation of Bone Marrow Macrophages (BMMs)

Fresh BMMs were flushed from the femur and tibia marrow of 5-week-old C57/BL6 female mice with α-MEM. The cells were slowly layered on a Ficoll-Hypaque gradient and then centrifuged at 440 g for 30 min at 4 °C. Cells at the gradient interface were classified as BMMs. BMMs were cultured in α-MEM containing 10% FBS and 30 ng/mL of M-CSF at 37 °C in a humidified atmosphere with 5% CO_2_.

### 4.3. Proliferation Viability Assay

RAW 264.7 cells and BMMs (10^3^ cells/well) were plated in 96-well plates in α-MEM with the indicated concentration (0, 0.5, 1, 2.5, 5, 10, 20 μM) of SFN. After 24 h of incubation, cell viability was measured using the CCK-8 assay according to the manufacturer’s instructions. Absorbance was measured at 450 nm. Cell viability was expressed as a percentage of the control.

### 4.4. Osteoclast Differentiation Assay and Fibrous Actin (F-actin) Ring Observation

RAW264.7 cells (10^4^/mL) and BMMs (10^5^/mL) were plated in 96-well plate with α-MEM containing 10% FBS, 50 ng/mL RANKL, and varying concentrations of SFN. M-CSF (30 ng/mL) was used for BMMs growth. Every 2 days, the medium was removed and substituted with fresh medium supplemented with the appropriate treatment reagents. Osteoclasts were observed after 5 days. Then, the cells were fixed with 4% PFA for 10 min and stained with the TRAP kit according to manufacturer’s instructions. TRAP-positive cells with >3 nuclei/cell were counted as osteoclasts under a light microscope.

After induction for 5 days, osteoclasts were fixed with 4% PFA for 10 min, permeabilized with 0.5% Triton X-100 for 5 min, and incubated with TRITC–phalloidin for 30 min at room temperature. After washing with PBS thrice, the cells were stained with DAPI for 30 s and washed again, and images were captured under an inverted fluorescence microscope.

### 4.5. Immunofluorescence Analysis

Appropriately treated cells were fixed with 4% paraformaldehyde, permeabilized with 0.5% Triton X-100 for 5 min, and blocked in 2.5% BSA for 1 h at room temperature. Then, the cells were incubated with a primary antibody (CTSK and LC3) at 4 °C overnight, washed twice with PBS, and then incubated with FITC-conjugated anti-goat IgG (1:100) for 1 h at room temperature. After washing with PBS thrice, the cells were incubated with TRITC–phalloidin for 30 min, and the nuclei were stained with DAPI for 30 s at room temperature. Then, the cells were observed and photographed under a fluorescence microscope.

### 4.6. Transmission Electron Microscopy (TEM)

The autophagosomes were observed by TEM (HT7700, Japan). Briefly, appropriately treated cells were collected, centrifuged, washed, and fixed with 2.5% glutaraldehyde in 0.1 M phosphate buffer. After dehydration with an ethanol gradient, the cells were embedded in resin and sectioned. Ultrathin sections were stained with saturated solutions of uranyl acetate and observed by TEM.

### 4.7. Quantitative Real-Time PCR (qRT-PCR) Analysis

Total cellular RNA was extracted by Trizol reagent. Then, mRNA was reverse-transcribed with the PrimeScriptTMRT reagent Kit with gDNA Eraser. qRT-PCR was conducted with SYBR Premix Ex Taq™ II. The primer sequences were designed (Table 1). Glyceraldehyde-3-phosphate dehydrogenase (GAPDH) was used to normalize the data and compared them to control values.

### 4.8. Western Blot Assay

Total proteins were extracted by radioimmunoprecipitation lysis buffer containing protease and phosphatase inhibitor, and the concentration was measured by the BCA assay. Western blot analyses were conducted after performing 10% SDS-PAGE, using 0.45 μm polyvinylidene fluoride membranes. Briefly, 30 μg of proteins were electrophoresed (60 V, 30 min; 110 V, 1 h) and subsequently transferred to the membranes (100 V, 53 min). After blocking with 5% BSA for 1 h at room temperature, the membranes were incubated with primary antibodies overnight at 4 °C. Following washing and incubating with horseradish peroxidase-conjugated anti-mouse/rabbit IgG for 1 h, the signals were detected by chemiluminescence (Bio-Rad, Singapore). The resulting protein levels were normalized to GAPDH level.

### 4.9. LPS-Mediated Calvarial Bone Erosion Experiment

The study was approved by the Ethics Committee of the Hospital of Stomatology at Wuhan University (approval numbers: S07918110A). All mice were raised in an SPF animal laboratory. C57/BL6 mice were divided into three groups (n = 3/group): control group, LPS group, and LPS plus SFN group. Mice were intraperitoneally injected with PBS or SFN (10 mg/kg body weight) the day before LPS treatment. Then, PBS or SFN was intraperitoneally injected 30 min before the daily LPS injection every other day for 6 days. LPS (10 mg/kg body weight) was injected at the midline of the calvaria located between the eyes and ears. All mice were sacrificed, and their calvarias were removed and fixed with 4% polyformaldehyde.

### 4.10. MicroCT Scanning and Analysis

Calvarias were scanned with a Skyscan 1176 microCT instrument (Broker, Kontich, Belgium) at a voxel size of 9 μm. The images were then reconstructed for the analysis. Bone histomorphometry analyses were performed with a microCT-associated software (Version 6.1, Scanco Medical AG, Wangen-Brüttisellen, Switzerland).

### 4.11. Bone Histological Analysis

After microCT, calvarias were sectioned for HE and TRAP staining. Briefly, calvarias were decalcified in 10% ethylenediaminetetraacetic acid, dehydrated in an alcohol gradient, embedded in paraffin, and sectioned. HE staining was conducted to show the erosion of bone. Then, parameters were evaluated including bone volume/total volume (BV/TV), trabecular number (Tb.N), trabecular thickness (Tb.Th), and trabecular separation (Tb.Sp). TRAP staining was performed according to manufacturer’s instructions to identify osteoclasts on the bone surface.

### 4.12. Immunohistochemical and Immunofluorescence Analysis

After deparaffinization in xylene, rehydration, antigen retrieval with a gastric enzyme, and block of endogenous peroxidase activity with 3% hydrogen peroxide, the sections were blocked with bovine serum albumin for 1 h and subsequently incubated with primary antibodies against CTSK or LC3 overnight at 4 °C. Subsequently, the sections were incubated with the Polink-2 plus polymer HRP detection system (immunohistochemical) or (FITC)-conjugated secondary antibodies (immunofluorescent analysis) for 1 h at room temperature. After washing with PBS thrice, the sections were observed and photographed under a microscope.

### 4.13. Statistical Analysis

Each independent experiment included three separate replicates. All quantitative values were expressed as mean ± SEM. All statistical data were analyzed with SPSS software (Chicago, IL, USA). Statistical comparisons were performed by one-way ANOVA followed by the Student–Newman–Keul test. Values with * *p* < 0.05 or ** *p* < 0.01 were considered statistically significance.

## 5. Conclusions

**Figure 7:**
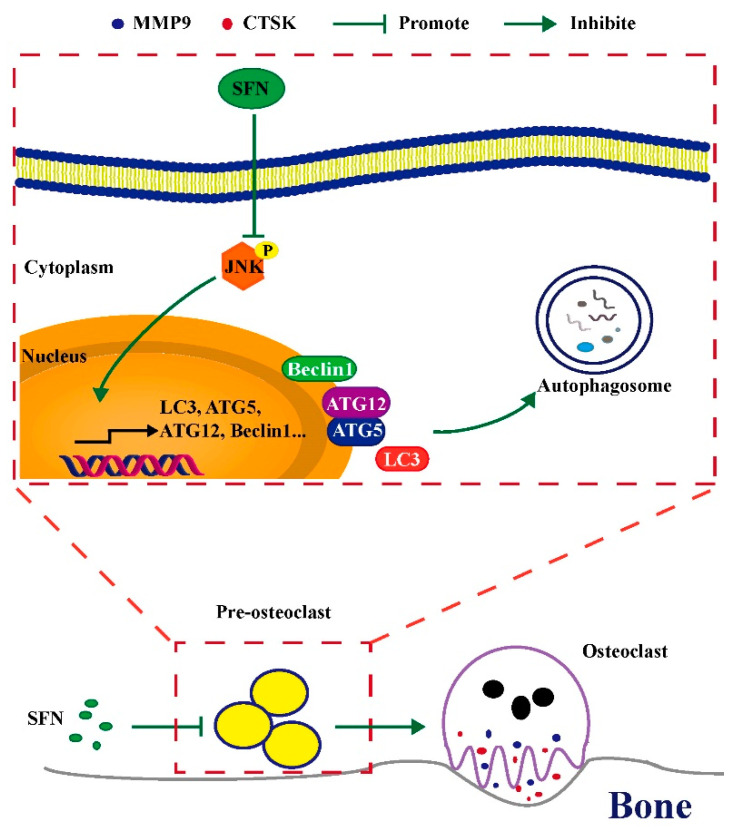
Schematic model of SFN-mediated anti-osteoclastogenesis. SFN inhibits the phosphorylation of JNK, decreasing the expression levels of autophagic proteins (Atg5, Atg12, Beclin1, and LC3). Subsequently, the level of osteoclastic autophagy is attenuated, which consequently suppresses osteoclastogenesis and bone erosion.

SFN could inhibit osteoclastogenesis by suppressing autophagy through the disruption of the JNK signaling pathway. (Figure 7). Therefore, SFN has great potential for the prevention and treatment of excessive osteoclastic bone destruction due to its ability to suppress this pathway. SFN administration might therefore be a new strategy for preventing and treating bone loss-related diseases.

## Figures and Tables

**Table 1:** Sequences of quantitative PCR primers.

Genes	Forward Primer	Reverse Primer
MMP9	CAAAGACCTGAAAACCTCCAAC	GACTGCTTCTCTCCCATCATC
CTSK	GCTTGGCATCTTTCCAGTTTTA	CAACACTGCATGGTTCACATTA
Atg5	AGTCAAGTGATCAACGAAATGC	TATTCCATGAGTTTCCGGTTGA
Atg12	GCCTCGGAACAGTTGTTTATTT	CAGTTTACCATCACTGCCAAAA
Beclin1	TAATAGCTTCACTCTGATCGGG	CAAACAGCGTTTGTAGTTCTGA
LC3	CTGTCCTGGATAAGACCAAGTT	GTCTTCATCCTTCTCCTGTTCA
GAPDH	GAPDH primer F and R were purchased from Sangon Biotech (Shanghai, China)	

## Data Availability

The data presented in this study are available manuscript.
